# Editorial: Options for Transition of Land Towards Intensive and Sustainable Agricultural Systems

**DOI:** 10.3389/fpls.2019.00346

**Published:** 2019-04-02

**Authors:** R. Millán, P. Schröder, A. Sæbø

**Affiliations:** ^1^Soil Conservation and Recuperation Unit, Environment Department, Centro de Investigaciones Energéticas, Medioambientales y Tecnológicas, Madrid, Spain; ^2^Research Unit for Comparative Microbiome Analysis, Helmholtz Zentrum München GmbH, Neuherberg, Germany; ^3^Norwegian Institute of Bioeconomy Research (NIBIO), Ås, Norway

**Keywords:** sustainable intensification, food production, biomass, soil amendments, fertilizers

Development of agricultural practices must be in accordance with the “Great Challenges” for the twenty-first century, for which soil and water protection are key factors. There is no single solution to these enormous challenges but efforts need to be made on all frontiers to feed our growing population and provide it with bio-based raw materials. Modern agriculture must adapt to fulfill these demands in a sustainable way. The main factors governing agroecosystem productivity are soil, water, balanced nutrients input, and a correct land management. In this context, adaptations to climate change must be addressed. Low water availability in Mediterranean arid and semi-arid conditions is one of the main threats for cropland. On the other hand, soils that today may be rather marginal because of cool summer climates, may become important for future food, feed, or industrial crops production if water is not limiting the production. The present research topic focuses on marginal land that can be considered for environmentally sound production of biomass or other marginal goods avoiding the use of good soil for this purpose.

Soils in many places are degraded to a stage where it takes long time to recuperate these resources, especially in contaminated areas. Thousands of sites in Europe have been declared as polluted with trace elements, and excluded from agricultural production. As a possible solution, Kidd et al. reports the adequate fertilization regimes; plant cropping patterns and plant microbial and fungal interactions and the biomass processed for Ni recovery. Mench et al. report a successful sunflower—tobacco crop rotation for copper removal, and Thijs et al. contribute results on the slow removal of Cd, Zn, and Pb from diffusely polluted soils by phyto-management using energy plants in a short rotation strategy.

It is a paradox that nutrients that may be very scarce in the near future, as P and N, are dispersed in far too large quantities, especially in areas with livestock production causing severe environmental problems. Resource depletion may also lead to price tensions with an impact on food security. Furthermore, some conventional agriculture practices represent a threat to sustainability of the long-term productivity. New methods, in line with principles of bioeconomy and circular economy models, spur the use of organic wastes as raw material for fertilizer and soil amendments. Ghaley et al. simulated soil organic carbon effects on winter wheat under different N-fertilizer amounts, using long term data from an experiment in Denmark. They concluded that agronomic productivity was enhanced by soil organic carbon build up when N-rates were in the range of 0–100 kg N ha^−1^, but not when N-fertilization was higher. Reichel et al. scrutinized the role of carbon source for the microbial storage or release of N compounds from arable land. They found that N immobilization by incorporated straw and sawdust may be more important for storage of excess N than microbial N immobilization over a growth period whereas pure lignin did not stimulate microbial N immobilization. Regarding biochar production from feedstocks, it is necessary to establish critical factors for their application as soil amendments. Marmiroli et al. found that the feedstock type determined biochar microstructure and elemental composition, which were linked to toxicity: biochar from animal origin were phytotoxic at lower concentrations than those from plant feedstock.

Synthetic fertilizers are annually 11.4 million tons of nitrogen and 1.1 million tons of phosphorus in EU-28. Meanwhile, between 118 and 138 million tons of biowaste are produced annually, but only about 25% is effectively recycled into compost and digestate. Fortunately, there is an increasing awareness that recycling of nutrients and organic matter is essential for creating sustainable food and non-food value chains. Energy crops and residues may be one option for more efficient use of biomass. Weiß and Glasner addressed how to sort out fractions that have negative impact on incinerators, by decreasing the chlorine content of the biomass of wheat chaff.

To prevent environmental pollution and ensure safety related to the uncontrolled application of inadequate amendments for agricultural purposes, updating of the regulations are being enforced on international level. In this context, a study investigating the effects of N sources and tillage practices on NH_3_ volatilization, grain yield and nutrient use efficiency (NUE) from paddy fields in central China is of high interest (Liu et al.). Furthermore, van Duijnen et al., found that N fertilizers affected wheat yield most but also pre-crops were important, and plants with N-fixing bacteria had a better effect on barley yield than mycorrhiza associated pre-crops. Improving fertility of marginal soils for the sustainable production of biomass is a strategy for reducing land use conflicts between food and energy crops as it is shown by Nabel et al. They demonstrate that the intercropping of legumes, can stimulate the yield of *S. hermaphrodita* on marginal soils for sustainable plant biomass production. Further on, Nabel et al. scrutinized the role of depot fertilization. This technique promoted a deep reaching root system of *S. hermaphrodita* seedlings with a dense root cluster around the depot-fertilized zone, resulting in 5-fold increased biomass yield.

A big problem is fodder, water and resource use for cattle growth. When forage production, feed intake, and animal performance of abandoned grassland before and after the common practice of rangeland grazing were compared, it became clear that extended spring grazing on abandoned grassland would improve lamb performance. Quantifying these aspects of reintroducing abandoned grassland into sheep farming gives both sheep farmers and land owners a knowledge basis for valuing the area in monetary terms and for decision-making (Steinshamn et al.).

New methods and technologies are needed, implementing remote sensing tools and drones, the use of precise methods and unmanned vehicles and transfer to smaller and smarter machines that are precisely guided. We must avoid destruction of soil structure, which encompasses a multitude of soil organisms important for soil functioning. Furthermore, crop rotation, intercropping, improved soil amendments, cultivar selection and irrigation methods need to be adapted for each specific situation. Finally, agricultural wastes must be investigated for their usability. Current research shows that we have many options and solutions that must be followed up and further developed to technology readiness (Schröder et al., 2018). In this context, poor, contaminated and dry soils may have to be re-activated in order to increase soil quality and soil potential for food, feed, and other biomass production (Mench et al.; Marmiroli et al.).

The current Frontiers research topic gives input to mobilization for increased food supply and shows that a multitude of actions are needed, and research and development is an important part. By showing that marginal and abandoned crop or grasslands have a value, and that techniques for sound production on such sites exist, such areas can give crucial contributions to biomass production. Future land use must embrace efficient production and utilization of biomass for improved economic, environmental, and social outcomes. New options open up a wide range of novel products and services across farming communities. A holistic approach is needed to identify common traits and at the same time enable the development and dissemination of production chains for sustainable intensification, which are adapted to the environmental and socioeconomic diversity situations.

## Author Contributions

All authors listed have made a substantial, direct and intellectual contribution to the work, and approved it for publication.

### Conflict of Interest Statement

The authors declare that the research was conducted in the absence of any commercial or financial relationships that could be construed as a potential conflict of interest.
